# Muscular involvement and tendon contracture in limb-girdle muscular dystrophy 2Y: a mild adult phenotype and literature review

**DOI:** 10.1186/s12891-020-03616-4

**Published:** 2020-09-01

**Authors:** Xuelin Feng, Jinlang Wu, Wenbiao Xian, Bing Liao, Songjie Liao, Xiaoli Yao, Weixi Zhang

**Affiliations:** 1 Department of Neurology, The First Affiliated Hospital, Sun Yat-sen University; Guangdong Provincial Key Laboratory of Diagnosis and Treatment of Major Neurological Diseases, National Key Clinical Department and Key Discipline of Neurology, No.58 Zhongshan Road 2, Guangzhou, 510080 China; 2 Laboratory of Electron Microscope, Zhongshan School of Medicine, Sun Yat-sen University, Guangzhou, 510080 China; 3 Department of Pathology, the First Affiliated Hospital, Sun Yat-sen University, Guangzhou, 510080 China

**Keywords:** LGM2Y, LAP1, Nuclear envelopathies, Tendon contracture, Case report

## Abstract

**Background:**

Limb girdle muscular dystrophy type 2Y (LGMD2Y) is a rare subgroup of limb girdle muscular dystrophy featuring limb-girdle weakness, tendon contracture and cardiac involvement. It is caused by the mutation of TOR1AIP1, which encodes nuclear membrane protein LAP1 (lamina-associated polypeptide 1) and comprises heterogeneous phenotypes. The present study reported a patient with a novel homozygous TOR1AIP1 mutation that presented with selective muscle weakness, which further expanded the phenotype of LGMD2Y- and TOR1AIP1-associated nuclear envelopathies.

**Case presentation:**

A 40-year-old male presented with Achilles tendon contracture and muscle weakness that bothered him from 8 years old. While the strength of his distal and proximal upper limbs was severely impaired, the function of his lower limbs was relatively spared. Muscle pathology showed dystrophic features, and electron microscopy showed ultrastructural abnormalities of disrupted muscle nuclei envelopes. Whole-exome sequencing showed a frameshift mutation in TOR1AIP1 (c.98dupC).

**Conclusion:**

We reported a novel mild phenotype of LGMD2Y with relatively selective distal upper limb weakness and joint contracture and revealed the heterogeneity of LGDM2Y and the role of the LAP1 isoform by literature review.

## Background

Limb-girdle muscular dystrophy (LGMD) is one of the most common muscular diseases featuring proximal muscle weakness [1]. However, some LGMD patients also present distal muscle weakness and tendon contracture, making it difficult to establish a diagnosis. LGMD2Y is a special subgroup of LGMD that presents distal weakness, joint contracture, restrictive lung disease and cardiac involvement in addition to limb-girdle muscle weakness [2]. LGMD2Y has been reported in Turkey [2], Australia [3], the USA [4] and Germany [5]. It is an autosomal recessive-inherited disease caused by a mutation in the torsinA-interacting protein 1 (TOR1AIP1) gene [2], which encodes lamina-associated polypeptide 1 (LAP1), a nuclear envelope protein [6]. It is a lamina-binding protein and is related to maintaining the nuclear membrane, and its defect is related to muscular dystrophy, dystonia and multisystem disorder [7].

The present study reported an adult Chinese pedigree with a novel homozygous TOR1AIP1 mutation, who presents joint contracture, distal weakness and proximal weakness with upper limb predominance. This pure muscular and tendon-involved presentation further expands the phenotype of TOR1AIP1-associated nuclear envelopathies.

## Case presentation

### Patient recruitment and consent

The study included a patient who was admitted to the Department of Neurology, the First Affiliated Hospital, Sun Yat-sen University, in February 2019. Written informed consent for participation was obtained from the patient. The study was approved under the guidelines of the Institutional Ethics Committee of the First Affiliated Hospital of Sun Yat-Sen University.

### Clinical features of LGMD2Y

**Fig. 1:**
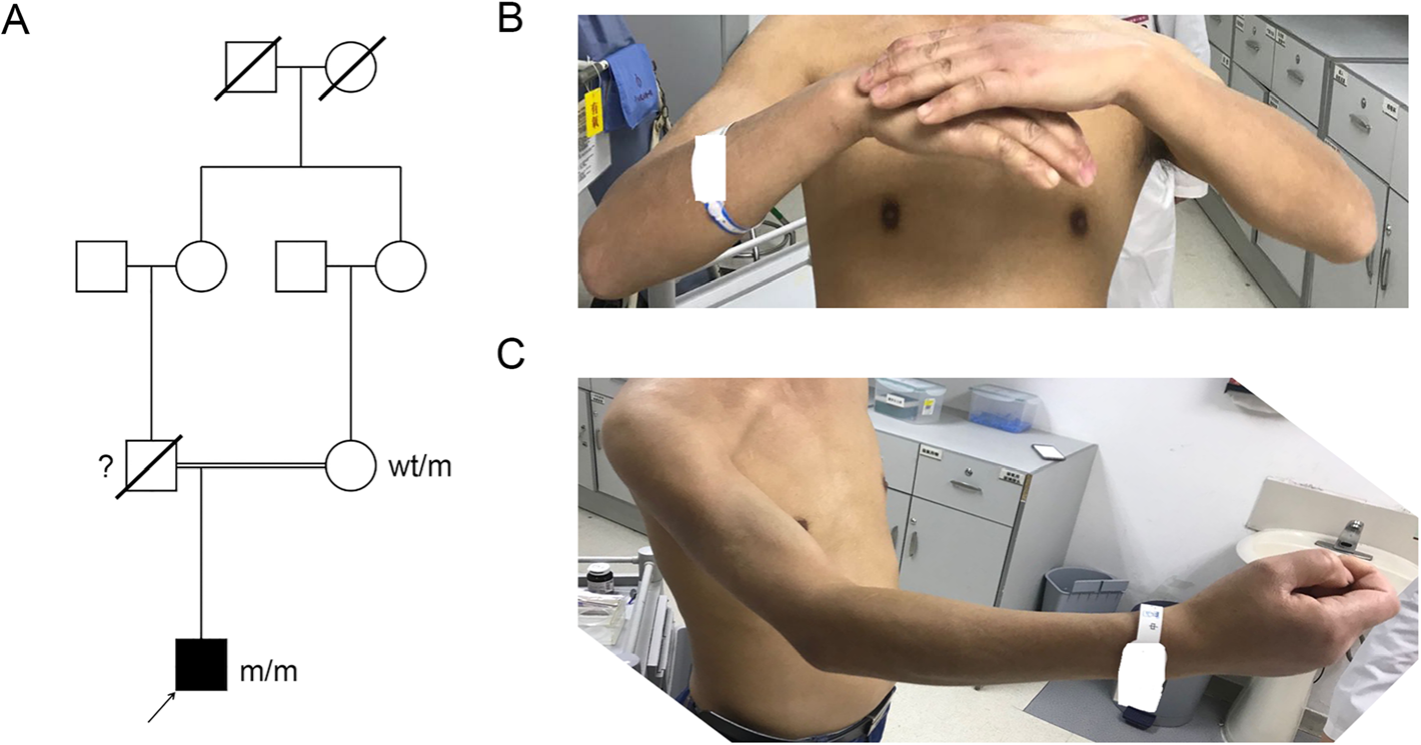
Family pedigree and clinical presentation of the present patient. (A) Family pedigree. DNA samples were obtained from the proband and his mother. They were tested by next-generation sequencing and Sanger sequencing, respectively. (B-C) Sign of upper limb weakness of the present patient. He could not extend his wrist and fingers (B). His proximal muscle of the upper limbs was atrophied (C)

The patient was a 40-year-old male from a consanguineous family in Northwest China who suffered from muscle weakness and tendon contracture (Fig. 1a). The patient complained of muscle weakness in the proximal lower limbs and upper limbs at 8 years old. He complained of disability in jumping or running fast in childhood. His distal upper limbs were preferentially impaired. He was unable to extend the wrist and fingers (Current Medical Research Council (MRC) score: 1, Fig. 1b). Gradually, the proximal upper limbs had progressive muscle weakness with preserved muscle strength in the lower limbs. At age 20 years, he was unable to raise the arms. Currently, the Gardner-Medwin and Walton modified scale of clinical severity score (GMW) [8] score is 1, and his 6-min walk test [9] result is 565 m. However, the strength of the upper limbs decreased severely with dominant proximal muscle atrophy (Fig. 1c). His Brooke Upper Extremity Rating Scale [10] was grade 5. The performance of the upper limb (PUL) [11] score of his shoulder, elbow and hand/finger was 6, 16 and 11, respectively. The strength of neck flexion was mildly decreased (MRC score: 4), while neck extension was spared.

Regarding tendon contracture, Achilles tendon contracture was revealed, while interphalangeal joints, elbow and spine were spared. Laboratory examination showed that creatine kinase levels were mildly elevated (230 U/L) and lactate dehydrogenase levels were normal. In the never conduction test, the conduction velocity and F wave were normal in each limb. In the electromyography test, no spontaneous potential was found, and several polyphasic motor unit potentials with short durations were documented.

### Pathological finding

**Fig. 2:**
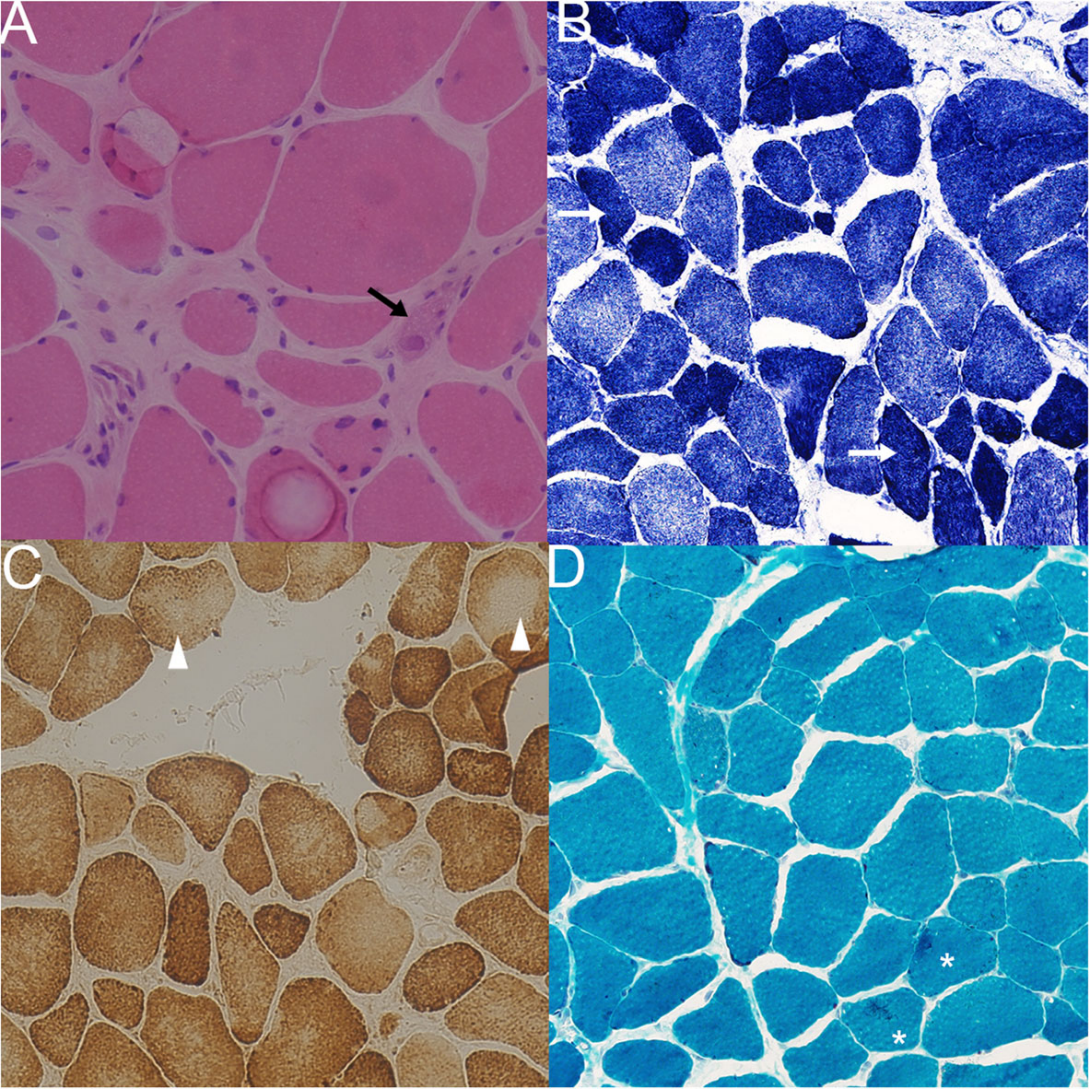
Histopathological findings of the present patient (magnification: 20 × 10). (A) Haematoxylin and eosin staining of the present patient. Muscle fibres were irregular and varied in size. Necrotic fibre (black arrow) with mild inflammatory infiltration was found. (B) Nicotine adenine dinucleotide tetrazolium reductase staining of the present patient. The internal architecture of some muscle fibres was damaged (white arrow). (C) Cytochrome C oxidase staining of the present patient. Reduced cytochrome C oxidase activity was found in some fibres (arrowhead). (D) Modified Gomori’s trichrome staining of the present patient. Aggregation in muscle fibre was found (*)

The muscle biopsy showed dystrophic features (Fig. 2a). Irregular and hypertrophic fibres were found. The fibrosis of the perimysium and endomysium was increased. Split fibres were observed. Some muscle fibres had increased internal nuclei. Degenerating and necrotic fibres with mild local inflammatory infiltration were observed. Disorganized muscle fibre structure was identified by nicotine adenine dinucleotide tetrazolium reductase (NADH-TR) staining (Fig. 2b). In addition, reduced cytochrome C oxidase activity was also found in some fibres (Fig. 2c). In modified Gomori trichrome staining, aggregation was found in a few muscle fibres (Fig. 2d). Oil Red O, periodic acid-Schiff, ATPase, dystrophin, dysferlin and major histocompatibility complex 1 staining were normal.

**Fig. 3:**
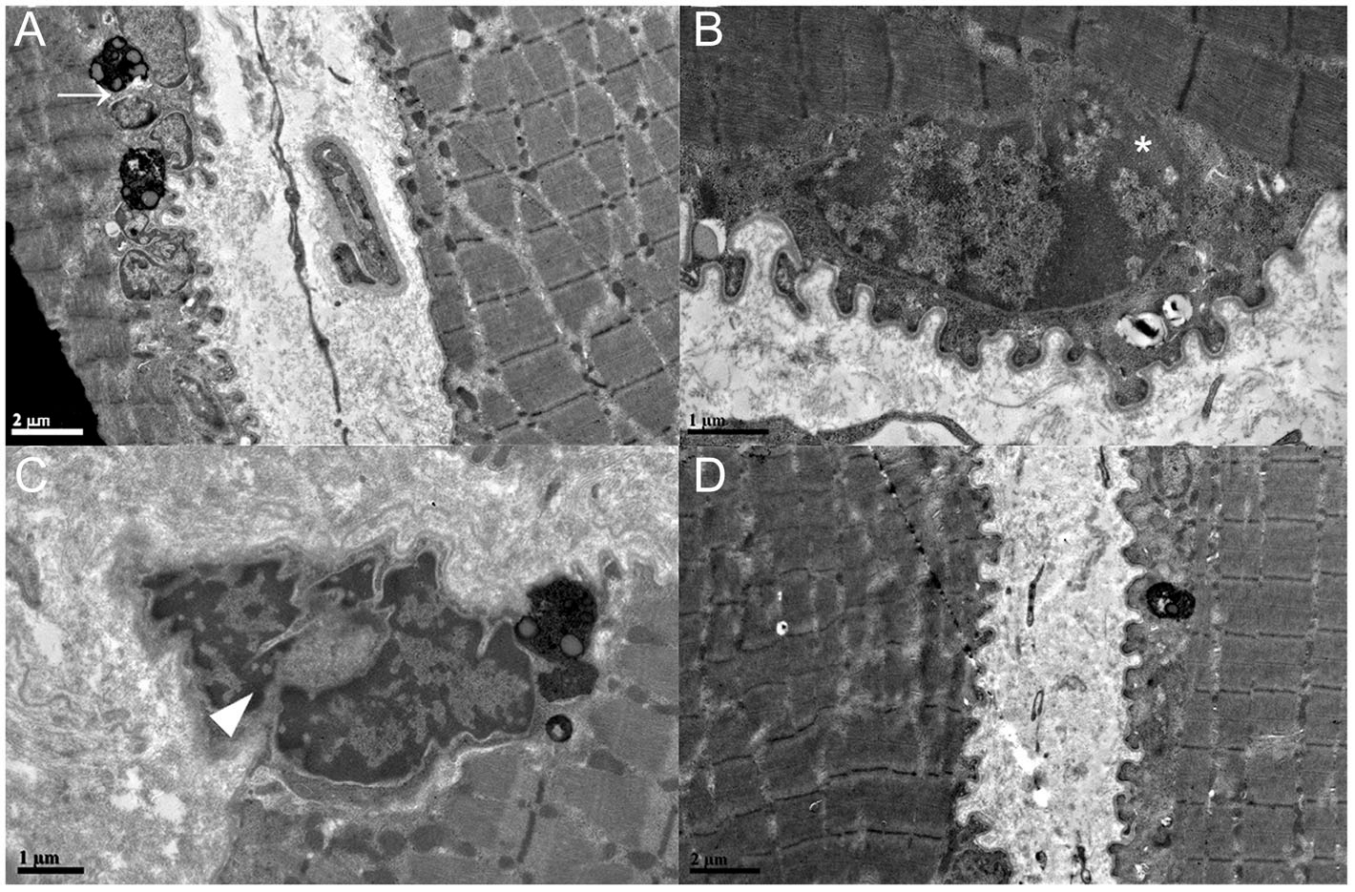
Ultrastructural findings of the present patients. (A) Multiple nuclei (arrow), chromatin condensation and segmented nuclei. (B) Nuclear membrane disruption and naked chromatin (*). (C) Chromatin condensation and broken nucleus (arrowhead). (D) Normal structure of sarcomeres

In transmission electron microscopy, ultrastructural abnormalities of muscle nuclei were found, including multiple nuclei, chromatin condensation, segmented nuclei and naked chromatin with a disrupted envelope (Fig. 3a-c). Increased lysosomes were identified in the subsarcolemmal area (Fig. 3d). The structure of the myofibril was spared, and no deposition was found.

### Genetic analysis

**Fig. 4:**
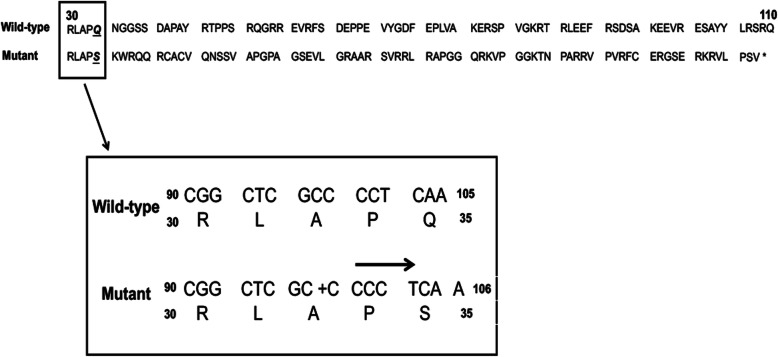
Genetic findings of the present patients. (A) c.98dupC mutation and corresponding amino acid alternation (p.Q35Sfs*74). Note the premature stop resulting from the frameshift in position 109

Whole-exome sequencing was used to establish a genetic diagnosis. It was performed on a NextSeq 500 (Illumina, Inc., USA). Burrows-Wheeler Aligner (version 0.7.10) was used for read mapping [12], and the Genome Analysis Toolkit (version 4.0.8.1) was used for adjustment and discovery of SNPs, insertions and deletions [13]. Annotation was carried out by Annovar (version: 2018-04-16) [14]. A novel homozygous mutation in TOR1AIP1 was found (c.98dupC, Fig. 4), leading to a frameshift mutation (p.Q35Sfs*74). Its frequency was examined in population databases, including the 1000 Genomes Project (https://www.internationalgenome.org/), the NHLBI Exome Sequencing Project (https://evs.gs.washington.edu/EVS/) and The Exome Aggregation Consortium (http://exac.hms.harvard.edu/). This mutant was classified as “likely pathogenic” according to the American College of Medical Genetics and Genomics standards (PSV1 + PM2) [15]. Sanger sequencing of the patient’s mother revealed the same mutation in TOR1AIP1 (a sample from the patient’s father was not available).

## Discussion and conclusion

TOR1AIP1-associated nuclear envelopathies comprise a spectrum of phenotypes ranging from a severe infantile form with multiple system involvement to a mild form of pure tendon and muscle involvement such as LGMD2Y. Our study presented an adult case with mild limb girdle involvement as well as distal muscle weakness due to a novel N-terminal frameshift mutation. This relatively mild adult phenotype further expands the understanding of genotype-phenotype correlation and provides diagnostic clues in neurological assessment for LGMD.

LGMD2Y is a rare subgroup of autosomal recessive LGMD, and only a few cases have been reported (Table 1) [2–5]. We reviewed the literature and summarized the phenotypes and genotypes. Most cases had disease onset in the first or second decade and insidiously progressed. The main clinical presentations include proximal weakness and atrophy of limbs and tendon contracture. The most common tendon contracture was contracture of the Achilles tendon (4/6) and interphalangeal joints (4/6). Other signs include rigid spine and kyphoscoliosis. Some of the patients presented with distal weakness and atrophy of limbs (4/8). Cardiac involvement (5/7) and restrictive lung disease (4/5) were common. Notably, four cases of dilated myocardiopathy have been reported, and most of them require heart transplantation or die due to heart failure [3, 5]. Only one muscle magnetic resonance imaging showed selective fatty infiltration in the medial and posterior parts of the thigh [4]. In contrast to a previous report, our case of LGDM2Y showed dominant impairment of the upper limbs, especially the muscle of the distal upper limbs. While he was still to retain the function of his lower limbs (6-min walk test: 565 m, better than the previous case and no impairment of distal lower limbs), his upper limbs were severely affected (PUL score: 33 and unable to extend wrist and finger). In addition, axial muscle weakness was also noticed (decreased neck flexion strength).

**Table 1:** Summary of limb-girdle muscular dystrophy 2Y in previous studies

	IV:5 [2]	IV:2 [2]	II:1 [3]	II:2 [3]	Unknown Diagnosis Network case [4]	Individual 1 [5]	Individual 2 [5]	Present case
**Sex**	Female	Male	Male	Female	Female	Male	Male	Male
**Developmental milestones of motor**	NA	NA	Delayed	NA	Not able to jump or run	Normal	Delayed	Normal
**Onset age of muscle weakness**	7y	17y	10y	10y	8y	between 8y and 31y	12y	8y
**Symptom**								
**Proximal muscle**	Limb-girdle weakness	Limb-girdle weakness	Limb-girdle weakness	Normal	Proximal weakness of lower limbs	Limb-girdle weakness	Limb-girdle weakness	Limb-girdle weakness, upper limbs predominace
**Distal muscle**	Normal	Severe distal weakness and atrophy	Weakness of foot dorsiflexion	Normal	Normal	Normal	Global muscle weakness at 16y	Unable to extend his wrist and finger
**Others**	–	–	Ptosis	Cardica failure and lethargy	Dysphagia of liquids and urinary incontinence	Multisystem anomalies and progeroid features	Multisystem anomalies and progeroid features	–
**Function of lower limbs**	Not able to rise from floor(29y)	Walk for 40 m(36y)	6-min walk test: 250 m(21y)	NA	Not able to walk for more than 25–50 ft.(14y)	NA	Mostly wheelchair-bound(16y)	6-min walk test: 565 m(40y)
**Disorder of joints and spine**								
**Interphalangeal joints**	Y	N	Y	NA	NA	Y	Y	N
**Achilles tendon**	N	Y	Y	NA	NA	N	Y	Y
**Spine disorder**	Rigid spine	Rigid spine	Thoracic kyphosis	NA	NA	N	kyphoscoliosis	N
**Others**	–	–	–	–	–	–	Mixed pectus carinatum and pectus excavatum	–
							Joint contracture of elbows and wrists	
**Cardiac symptoms**	Normal	Mild diastolic and systolic dysfunction	Dilated cardiomyopathy	Dilated cardiomyopathy	NA	Dilated cardiomyopathy	Dilated cardiomyopathy	No related complainment
		Ventricular extrasystoles	Heart transplantation	Heart transplantation		Implantable cardioverter defibrillator implantation	Died at 16y due to heart failure	
**FEV1%**	43%	63%	Normal	NA	Reduced	NA	NA	NA
**Gene**	c.186delG (homo)	c.186delG (homo)	c.127delC	c.127delC	NA	c.945_948delCAGT	c.724delG	c.98dupC (homo)
			c.1181 T > C	c.1181 T > C		c.1331G > C	c.649G > T	
**CK (U/L)**	89	483	Normal	NA	10,000	NA	45	338
**Muscle MRI**	NA	NA	NA	NA	Bilateral symmetric atrophy of the medial and posterior compartmen in thigh	NA	NA	NA
					No atrophy in calf			
**Muscle pathology**								
**myopathic feature**	Y	Y	Y	NA	Y	NA	Y	Y
**inner nuclei**	Y	Y	Y		N		Y	Y
**angular fibre**	N	Y	Y		N		N	Y
**Split fiber**	N	N	Y		N		N	Y
**Others**	–	Nuclear clumps	–		Perimysial and endomysial lymphocytic inflammation		–	Aggregation
		Type 2 fibres predominated			Positive MHC-1 and C5b9 staining			Reduced oxidative enzymes activity in Type 2 fibers
		Subsarcolemmal vacuolar change			Core-like structure			
					Rimmed vacuoles			
**Ultrastructural change**								
**envelope disruption**	NA	Y	Not mentioned	NA	Y	NA	NA	Y
**Spare Sarcomere**		Y	Y		Core-like structure			Y
**Others**		–	–		Autophagic debris			Increased subsarcolemmal lysosome
					basal lamina duplication			

* NA:Not available; FEV1:First second forced expiratory volume; MHC:major histocompatibility complex

In muscle pathology, most of the reported LGMD2Y cases had myopathic changes under a light microscope. Necrosis with inflammatory infiltration was uncommon but was also reported in a rapidly exacerbated case [4]. Ultrastructural alternation was mostly restricted in nuclei varying from chromatin condensation to envelope disruption and naked chromatin. Sarcomeric architecture was always spared. The specimens of the present patients also showed myopathic changes on light microscopy (Fig. 2a) and envelope disruption on electron microscopy (Fig. 3).

In addition to muscular dystrophy and cardiac involvement, mutation of TOR1AIP1 also resulted in dystonia [16], cerebellar atrophy [16], progeroid features and multisystem disorder [5, 17], which would also present with muscular dystrophy [5]. The heterogeneity of TOR1AIP1-related envelopathies may result from the two different isoforms of LAP1 in humans, LAP1B and LAP1C [18]. The absence of LAP1B was related to muscular dystrophy [2], and the loss of LAP1C was related to multisystem syndrome [5, 16, 17].

Interestingly, the muscular dystrophy cases reported by Ghaoui et al. [3] was a heterozygote who had a LAP1C-affected allele and LAP1C-preserved allele. This may explain why their symptoms were restricted to skeletal muscle and cardiac muscle but also had more severe cardiomyopathy. In addition, the present case was less affected than other LAP1C-preserved patients considering his present lower limb function (GMW 1 vs. 5–8, compared with the case reported by Kayman-Kurekci et al. [2]). The mechanism of heterogeneity among muscular dystrophy cases is still not clear. The mutation of all reported muscular dystrophy cases was in the first exon, which encodes the intranuclear part of LAP1. Most of them were in the beginning of the peptide (position 35–62), which may regulate the translation or post-translation modification of LAP1C. By regulating LAP1C, the difference in TOR1AIP1 mutation resulted in the heterogeneous presentation of muscular dystrophy cases.

LAP1 is a nuclear envelope-associated protein [6] that contributes to nuclear envelope maintenance. As a result of nuclear envelope alteration, LGMD2Y shares some common points with Emery-Dreifuss muscular dystrophy, which is also caused by mutations in nuclear envelope proteins, such as emerin [19] and nesprin [20]. Both of them featured limb girdle muscular dystrophy, joint contracture and cardiac involvement [21]. However, the mechanism of such a connection between this phenotype and nuclear envelope alteration is still not clear. Interestingly, mutations in other nuclear envelope proteins, such as nesprin-1 [22], also resulted in central nervous system disorders similar to LAP1. In addition, the heterogeneity of nesprin-1 was also related to the mutation position and corresponding isoform [23]. The heterogeneity of nuclear envelope proteins and their connection with isoforms deserve further investigation.

Our study reported a case of LGMD2Y with relatively selective distal upper limb weakness and joint contracture. After our review of reported TOR1AIP1 mutation cases, we revealed the heterogeneity of LGMD2Y and the role of LAP1C.

## Data Availability

The datasets used and/or analysed during the current study are available from the corresponding author on reasonable request.

## Abbreviations

GMW: Gardner-Medwin and Walton modified scale of clinical severity score
LAP1: lamina-associated polypeptide 1
LGMD: Limb-girdle muscular dystrophy
MRC: Medical Research Council
PUL: Performance of upper limbs
TOR1AIP1: torsinA-interacting protein 1
